# The Role of the Immune System in Triplet Repeat Expansion Diseases

**DOI:** 10.1155/2015/873860

**Published:** 2015-03-22

**Authors:** Marta Olejniczak, Martyna O. Urbanek, Wlodzimierz J. Krzyzosiak

**Affiliations:** Department of Molecular Biomedicine, Institute of Bioorganic Chemistry, Polish Academy of Sciences, Noskowskiego 12/14, 61-704 Poznan, Poland

## Abstract

Trinucleotide repeat expansion disorders (TREDs) are a group of dominantly inherited neurological diseases caused by the expansion of unstable repeats in specific regions of the associated genes. Expansion of CAG repeat tracts in translated regions of the respective genes results in polyglutamine- (polyQ-) rich proteins that form intracellular aggregates that affect numerous cellular activities. Recent evidence suggests the involvement of an RNA toxicity component in polyQ expansion disorders, thus increasing the complexity of the pathogenic processes. Neurodegeneration, accompanied by reactive gliosis and astrocytosis is the common feature of most TREDs, which may suggest involvement of inflammation in pathogenesis. Indeed, a number of immune response markers have been observed in the blood and CNS of patients and mouse models, and the activation of these markers was even observed in the premanifest stage of the disease. Although inflammation is not an initiating factor of TREDs, growing evidence indicates that inflammatory responses involving astrocytes, microglia, and the peripheral immune system may contribute to disease progression. Herein, we review the involvement of the immune system in the pathogenesis of triplet repeat expansion diseases, with particular emphasis on polyglutamine disorders. We also present various therapeutic approaches targeting the dysregulated inflammation pathways in these diseases.

## 1. Introduction

A number of human inherited neurological disorders are caused by the expansion of trinucleotide repeats in specific, functionally unrelated genes [1, 2]. Based on the localization of repeats in transcripts, triplet repeat expansion diseases (TREDs) are classified into coding and noncoding repeat expansion disorders. Mutant proteins and transcripts are toxic factors in a group of polyglutamine (polyQ) diseases, for example, Huntington's disease (HD), that are caused by the expansion of CAG repeats in open reading frames (ORFs) of implicated genes. An RNA* gain-of-function* mechanism is thought to be responsible for noncoding repeat expansion disorders, including myotonic dystrophy type 1 (DM1) and fragile X-associated tremor ataxia syndrome (FXTAS). The age of onset and the severity of symptoms correlate with the size of the expansion, with a threshold of approximately 40 CAG repeats in most polyQ diseases and more than 50 CTG/CGG repeats in nontranslated repeat disorders. The functions of the proteins, the main sites of pathogenesis, and the characteristics of the clinical features are presented in Supplementary Table 1 (see Supplementary Material available online at http://dx.doi.org/10.1155/2015/873860). Although these late-onset diseases are characterized predominantly by neurological manifestations, several peripheral tissues show abnormalities in morphology and function, for example, skeletal muscles, hepatocytes, kidney, and blood cells [3, 4].

The common feature of most TREDs is the loss of neurons in specific brain regions accompanied by reactive gliosis and astrocytosis, which may suggest involvement of inflammation in pathogenesis. The inflammatory response on the CNS level is reflected by the interplay between neurons, microglial cells, and astrocytes, and the effects observed in the CNS may be further modulated by blood cells that fulfill immunological functions in the periphery.

The innate immune response plays a key role in recognition of pathogen-associated molecular patterns (PAMPs), exogenous signals derived from microorganisms (e.g., unmethylated CpG DNA, viral RNA, and 5′-triphosphate RNA, as well as lipoproteins, surface glycoproteins, and membrane components, e.g., lipopolysaccharide (LPS)). Cells release also endogenous danger signals, known as damage-associated molecular pattern molecules (DAMPs) that alert the innate immune system in response to stress. PAMPs as well as DAMPs, such as nucleic acids, ATP, and aggregated or misfolded proteins, are recognized in cells by conserved sensors, known as pattern-recognition receptors (PRRs). These include, for example, Toll-like receptors, an IFN-inducible dsRNA-activated protein kinase (PKR), retinoid acid-inducible gene I- (RIG-I-) like receptors (RLRs), AIM2 like receptors (ALRs), and NOD-like receptors (NLRs) that trigger inflammasome assembly and caspase activation. Stimulation of PRRs leads to activation of intracellular signaling pathways, including transcription factors (e.g., nuclear factor-*κ*B (NF-*κ*B), AP-1 and IFN regulatory factors (IRFs)), and the synthesis of signaling molecules, such as cytokines, chemokines, and immunoreceptors [5]. Persistent stimulation of immune system and synthesis of cytokines such as IL-6, IL-8, or TNF-*α* can contribute to degeneration of cells and disease progression.

In neurodegenerative diseases, such as Alzheimer's disease (AD), Parkinson's disease (PD), and amyotrophic lateral sclerosis (ALS), the role of inflammation in neurodegeneration is well documented [6–8]. Similarly, in TREDs, chronic stimulation of the immune system by a mutant protein/transcript may play an important role in disease progression. A number of immune response markers, including elevated cytokine, reactive oxygen species (ROS), and nitric oxide (NO) levels, activation of caspases, and changes in gene expression have been observed in the blood and CNS of patients and mouse models. Some of these effects are even observed in the premanifest stage of the disease and may have diagnostic significance [9, 10]. It seems that ageing and environmental factors, such as infections, may influence and modulate this response, thus affecting the development of the disease [11, 12].

In this review, we present the current state of knowledge regarding the role of the immune system in the pathogenesis of TREDs. We discuss the topic from the perspective of not only the toxic proteins but also mutant transcripts, which may be an important and overlooked factor participating in this response. We also present therapeutic approaches targeting toxic RNAs and proteins that trigger pathological effects and induce various immune responses.

## 2. Inflammation in Polyglutamine Diseases

Currently, there are nine known inherited neurodegenerative disorders caused by the expansion of CAG repeats within the coding region of associated genes. These include HD, spinocerebellar ataxias types 1, 2, 3, 6, 7, and 17 (SCA), spinobulbar muscular atrophy (SBMA), and dentatorubral-pallidoluysian atrophy (DRPLA). The accumulation of polyglutamine-rich proteins that affect various cellular functions and cause selective neurodegeneration in specific brain regions is a common feature of polyQ diseases [13–15]. Most studies have focused on protein toxicity in the disease pathomechanism, but recent findings demonstrate that mutant transcripts also contribute to the disease* via* an RNA* gain-of-function* mechanism [16, 17]. This mechanism was initially described for noncoding repeat expansion disorders, such as myotonic dystrophy type 1, FXTAS, or myotonic dystrophy type 2 (DM2), reviewed in [18]. The RNA-mediated mechanism assumes that nuclear foci formed by mutant CAG-containing transcripts sequester specific RNA-binding proteins, leading to loss of their normal function.

Immune activation in polyQ diseases is found both in the central nervous system and in the blood (peripheral level) of patients and mouse models of the diseases. The crosstalk between these levels as well as between the innate and adaptive immune responses needs to be better recognized. Currently, most of the information concerning the immune response in polyQ diseases refers to Huntington's disease [19], but it is unclear whether HD is unique in this respect. There are still many unresolved questions concerning the role of the immune response in the pathogenesis of polyQ diseases. What is the contribution of the toxic entities, that is, proteins and transcripts, in the stimulation of the immune system? Are the changes in the immune system the cause or the consequence of neurodegeneration? What is the role of aging and exposure to environmental factors, such as infections (priming), in the pathogenesis? Is the immune system a good target for therapeutic interventions for polyQ diseases? We hope that the answers to at least some of these questions can be found in this review.

### 2.1. Inflammation in HD-CNS Level

Huntington's disease, the most common of the polyQ disorders, is caused by expansion of CAG repeats in exon 1 of the* HTT* gene, which encodes the huntingtin protein (The Huntington's Disease Collaborative Research Group, 1993). In the healthy population, the number of CAG repeats lies between 6 and 35 CAG units. Alleles with repeat lengths from 36 to 39 exhibit reduced penetrance, whereas forty or more repeats are fully penetrant and associated with the development of HD [20–22]. Selective loss of neurons in the striatum and cortex leads to progressive movement abnormalities, dementia, and eventually death, typically in the fourth or fifth decade of life. Mutant huntingtin is widely expressed, and its posttranslationally modified versions, frameshifting products [23, 24] or N-terminal cleavage fragments, may form toxic aggregates in cells [25]. The presence of a mutant protein leads to many abnormalities, such as mitochondrial dysfunction and oxidative stress, ubiquitin-proteasome system dysregulation, chaperone protein and autophagy inhibition, synaptic dysfunction, glutamate-induced excitotoxicity, and transcriptional dysregulation [12, 26, 27].

Mutant HTT is expressed in immune cells at high levels, and persistent stimulation of the immune system at the CNS level is manifested by microglia activation and reactive gliosis [26, 28]. Microglia (the macrophages of the CNS) surrounded by astrocytes and neurons are the major resident immune cells in the brain and serve as the frontline defense of the innate immune system. Under physiological conditions, microglia play roles in the programmed elimination of neural cells during development and in maintaining their survival by removing toxic cellular debris [29]. In response to a stimulus, microglia proliferate, migrate toward an immune stimulus, and induce a cascade of proinflammatory cytokines (e.g., IL-6, IL-12, TNF-*α*, and IL-1*β*). These, in turn, lead to caspase activation, changes in intracellular calcium levels, and free radical production. Excessive stimulation of these pathways may lead to neurodegeneration [30].

The microglia activation observed in postmortem HD brain tissue [31] is also detectable in the presymptomatic stage in HD gene carriers [9] and mouse models [27, 32]. This activation increases over the duration of the disease and correlates with the severity of disease progression [33, 34], suggesting a close relationship between microglial activation and neuronal death [26]. Microglia that express mutant HTT produce increased levels of proinflammatory cytokines, including IL-6, IL-8, and TNF-*α* [10], neurotoxic kynurenine metabolites [35], and have an impaired response to brain injury and migration to chemotactic stimuli [36]. Massive transcriptional induction of several chemokines, including monocyte chemoattractant protein-1 (MCP-1) and murine chemokine (KC), was detected in mouse neuroblastoma cells expressing mutant HTT [37]. This upregulation is explained by HTT-induced, mild proteasomal dysfunction and activity of the NF*κ*B transcription factor in neuronal cells.

Mutant huntingtin also disturbs the normal functions of other glial cells, mainly astrocytes [4, 38] that play many important roles in amino acid, nutrient, and ion metabolism in the brain, maintaining homeostasis at the synapse, regulating neuronal signaling, and protecting neurons from oxidative damage. Reactive astrocytes observed in brains of patients with HD are characterized by hypertrophy and upregulation of several molecules including GFAP, S100B, iNOS, and NF*κ*B. The role of astrocytes in inflammation is of great importance because reactive gliosis even occurs in HD models that do not express mutant HTT in neurons [38]. In addition, LPS-induced activation of proinflammatory cytokines in the brain was not observed in a mouse model that expressed mutant HTT in neurons (N171-82Q), but not in glial cells [39]. Hsiao et al. reported that mutant huntingtin enhanced the activity of I*κ*B kinase (IKK), leading to enhanced activation of transcription factor NF*κ*B in astrocytes of patients and mouse models of HD, but not in microglia and neurons. Such an IKK-NF*κ*B-mediated immune response leads to upregulation of inflammatory genes, caspase 3 activation, and neuron apoptosis. In another study using a mouse model of HD (R6/2 brains), it has been shown that effector molecules, such as caspases [40], iNOS [41], and proinflammatory nitric oxide, contribute to the observed astrogliosis and apoptosis in neighboring cells [40, 42]. Caspases are proteases that play essential roles in apoptosis. In the late presymptomatic stage of HD, mutant huntingtin-induced toxicity results in caspase-1 activation and IL-1*β* production. As disease progresses, caspase-3 is upregulated. This picture is further complicated by the fact that huntingtin is itself a substrate of caspases 1 and 3 [43–45], and its cleavage precedes neurodegeneration in HD [46]. Some evidence indicates that the complement system, which connects the innate and adaptive immune responses (e.g., C3 and C9 factors), is upregulated in brains of HD patients [47]. However, research on the involvement of the adaptive immune system in HD is still in its infancy.

**Figure 1:**
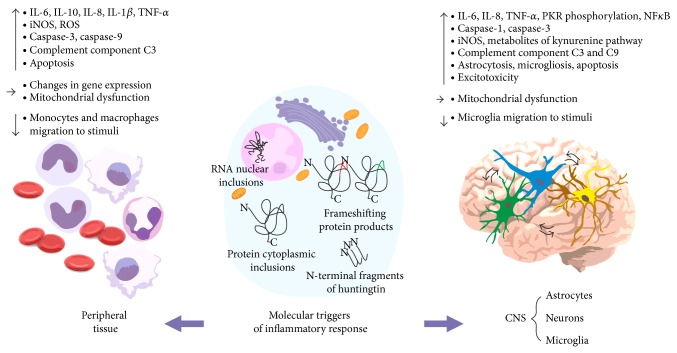
Inflammation in Huntington's disease. The mutant HTT transcript and protein are expressed in many cell types, including neurons, astrocytes, and blood cells of HD patients. The pathogenic effect may be triggered by expanded CAG repeat hairpins, cytoplasmic protein aggregates, N-terminal fragments of huntingtin, or toxic frameshifting products, and so forth. It is currently not clear what pathways are primarily involved in inducing the inflammatory response observed in the CNS and peripheral immune system. This effect is observed in the brain and peripheral tissues, indicating crosstalk in the signaling between distant tissues. The observed immune effects include elevated cytokine levels, caspase pathways activation, induction of apoptosis, dysregulation of gene expression, or decreased immune cell migration.

Taken together, the results of numerous studies show that immune system activation contributes to the neurodegeneration observed in HD and that the interplay between neurons, astrocytes, and microglia is responsible for these effects at the CNS level (Figure 1).

### 2.2. Inflammation in HD-Peripheral Level

Growing evidence supports the role of the peripheral immune system in HD pathogenesis. The immunological effects observed in the blood of patients and mouse models of HD are similar to those in the CNS and appear long before neurological symptoms (Figure 1). It has been shown that the level of mutant HTT in leukocytes increases with disease progression [48] and may act as a chronic stimulator of these cells. Elevated cytokines, for example, IL-6, IL-8, IL-4, IL-10, TNF-*α*, and IL-1*β* [10, 34], and chemokines, for example, eotaxin-3, MIP-1*β*, eotaxin, MCP-1, and MCP-4 [49], were observed in the plasma of HD patients and mouse models, such as YAC128 [10, 50], R6/2, and* Hdh* [10, 39] (Supplementary Table 2). Surprisingly, in the BACHD mouse model, the authors did not observe elevated cytokine levels. Monocytes and macrophages from patients with HD are hyperreactive in response to IFN*γ*/LPS stimulation, producing increased levels of IL-6, IL-8, and TNF-*α* [10, 51]. The TNF-*α* and IL-1*β* levels were also elevated in the serum and liver of mouse models after stimulation with LPS [39]. Interestingly, this hyperactivity of myeloid cells was abrogated by silencing HTT expression with siRNA [51], which suggested that immune cell activation was caused by a cell-autonomous effect of mutant HTT expression, rather than a secondary response to other extracellular factors. Furthermore, a significant association between CAG repeat length and the level of TNF-*α* produced by HD monocytes was observed [51], suggesting direct involvement of mutant HTT in triggering these effects. Träger et al. demonstrated that mutant huntingtin influenced the activity of the NF*κ*B transcription factor. Altered transcription of NF*κ*B target genes results in increased cytokine (IL-6 and TNF-*α*) production by immune cells. These results are consistent with those obtained by Hsiao et al., showing astrocyte-mediated IKK-NF*κ*B-dependent inflammation in brains [39]. The other dysfunctions observed in the blood of HD patients and mouse models include increased apoptosis [52], autophagy and caspase activation [53], transcriptional dysregulation [54, 55], and elevated levels of mitochondrial dysfunction markers [56]. Additionally, the kynurenine/tryptophan ratio, which is an indicator of ongoing inflammation, is elevated in the serum of HD patients and correlates with disease progression [35, 57].

Thus, peripheral immune system activation reflects the processes observed in the CNS; however, the direct role of these systems in the pathogenesis of HD must be better recognized.

### 2.3. Inflammation in Other PolyQ Diseases

Toxic transcripts and proteins containing polyglutamine tracts are common factors that may trigger pathogenic pathways in polyQ disorders. Several polyQ proteins, such as huntingtin, ataxin-3, ataxin-7, AR, and atrophin-1, are substrates for caspases. Truncated fragments of these proteins are more toxic than their full-length forms; therefore, they play crucial roles in the pathogenesis of each disease [58–60]. However, whether these toxic protein fragments induce immune responses similar to those for HD must be determined. In addition, during the translation of mutant HD and SCA3 transcripts, chimeric polyQ/polyAla proteins may be formed due to ribosomal frameshifting, thus increasing the variety of toxic entities [23, 24, 61]. The role of the immune response in the pathogenesis of other polyQ diseases is less explored than in the case of HD. The existing data are incomplete and limited mainly to spinocerebellar ataxia type 3 (SCA3), known also as Machado-Joseph disease (MJD), which is caused by expansion of CAG repeats in exon 10 of the* ATXN3* gene [62]. The CAG repeat length normally varies from 10 to 51, with 55–87 CAG repeats being reported to associate with the disease [2, 63]. Ataxin-3 is an ubiquitously expressed enzyme that functions in the proteasomal protein degradation pathway and in transcription regulation (Supplementary Table 1). Toxic intracellular aggregates of mutant ataxin-3 are observed in neurons from different regions of the brains of SCA3 patients and in cell and animal models of SCA3 [14, 64].

Studies on cell lines and brain tissues confirmed the involvement of inflammatory processes in the pathogenesis of SCA3 [65–67]. The authors identified genes directly or indirectly involved in the immune response that were upregulated in a mutant ataxin-3-expressing rat cell line (SCA3-Q70). These genes included endopeptidase matrix metalloproteinase 2 (*MMP-2)*, transmembrane protein amyloid precursor protein* (APP)*, interleukin-1 receptor-related Fos-inducible transcript* (Fit-1S)*, and cytokine stromal cell-derived factor 1*α* (*SDF1α*). Increased expression of the corresponding (MMP-2 and SDF1) or associated proteins, including anti-inflammatory interleukin-1 receptor antagonist (IL-1ra), the proinflammatory cytokine IL-1*β*, and the proinflammatory chemokine SDF1, was also demonstrated in human SCA3 pontine neurons. Evert et al. confirmed these results in their later study and demonstrated that in addition to the Fit-1S and IL-1ra cytokines, IL-6 and two cytokine-inducible transcription factors (C/EBP*β* and C/EBP*δ*) were also upregulated in mutant ataxin-3-expressing cell lines and pontine neurons of SCA3 patients [66]. The presence of nuclear inclusions is not a prerequisite for these transcriptional changes because increased expression of cytokines was observed in neurons with and without inclusions [67]. The identified activation of inflammatory pathways corresponds well with the observed neurodegeneration [65, 66]. Immunostaining performed on human brain tissues with the use of microglial and astrocytic markers (CD68 and GFAP, resp.) has shown increased numbers of activated microglial cells and reactive astrocytes in the pons of SCA3 patients. Microglial dysfunction and reactive astrocytosis were also observed in other SCAs [68–70].

Expanded ataxin-3 and ataxin-7 induce the apoptotic death of cultured cerebellar neurons by upregulation of the proapoptotic proteins Bax and PUMA and downregulation of Bcl-x_L_ (an antiapoptotic protein) [71–73]. Mutant ataxin 7 decreases the nuclear translocation of NF-*κ*B p65 and impairs NF-*κ*B activity by inhibiting proteasome activity in cerebellar neurons, leading to reduced Bcl-x_L_ expression, caspase activation, and cerebellar neuronal death. Moreover, the misfolded androgen receptor protein in the cytosol induces the Bax-dependent apoptotic cascade that is initiated by the JNK signaling pathway in cultured primary neurons from mice [74].

The role of the polyQ tract in triggering the immune response is still unclear. A SCA3 gene trap mouse model that expresses a truncated N-terminal region of the endogenous mouse ataxin-3 protein was generated to study the pathomechanism of SCA3. Despite the fact that the C-terminal region, which contains the polyQ tract, is missing, homozygous mutant mice still develop neurological symptoms and prematurely die. In contrast to observations of an HD mouse model [10], the levels of circulating cytokines were unchanged but showed high interindividual variability. The only observation that indicated some changes in the immune system, although unexplained by the authors, was the increased number of granulocytes and decreased number of B cells at 12 months [75].

The results described above confirm the involvement of the immune response in the pathomechanism of polyQ diseases, especially HD. However, the molecular mechanism and molecular triggers of these signaling cascades are still unknown.

## 3. RNA as a Trigger of the Immune Response

Most studies on the pathogenesis of polyQ diseases have traditionally focused on protein-based mechanisms of toxicity. However, growing evidence suggests that mutant transcripts may also play an important role in neurodegeneration [76–82], as was shown for untranslated trinucleotide diseases [79, 83, 84]. In contrary to normal transcripts, where CAG repeats either form unstable hairpins or do not form such structures at all, expanded repeats of mutant transcripts fold into more stable hairpin structures, which may interfere with normal cellular processes [85]. Such abnormal endogenous RNAs may serve as PAMPs recognized by pattern recognition receptors in cells. It cannot be excluded that stem-loop structures formed by mutant transcripts, products of bidirectional transcription, alternatively spliced transcripts, and RNAs released from necrotic cells are recognized as “non-self” molecules by cellular sensors of foreign RNA. In the next step, the activation of signal transduction pathways (e.g., MyD88, TRIF, NF-*κ*B, IRFs) may lead to the production of effector molecules such as cytokines and reactive oxygen species (ROS) that amplify the immune response and recruit additional immune cells.

There are at least 7 cytoplasmic and endosomal sensors of foreign RNA, and these include IFN-inducible dsRNA-activated protein kinase (PKR) [86], 2′-5′-oligoadenylate synthetase (OAS) [87], retinoic acid-inducible gene I (RIG-I), melanoma differentiation associated gene-5 (MDA5), and Toll-like receptors (TLR3, TLR7, and TLR8) [88, 89]. These sensors are highly expressed in cells that play important roles in innate immune responses, such as macrophages and microglia.

Activation of the PKR and OAS signaling pathways by long dsRNA results in the general inhibition of protein synthesis and the degradation of cellular RNA, respectively. It has been shown that PKR binds CUG repeat-containing DM1 transcripts* in vitro* in a length-dependent manner (with a minimal length of 15 CUG repeats) and is activated by pathologically expanded repeats [84]. CUG repeats cause stress in DM1 cells through the PKR-phospho-eIF2*α* pathway and inhibits translation of mRNAs associated with cytoplasmic stress granules (SGs) [90].

Similarly, mutant huntingtin mRNA also binds PKR in human brain tissue extracts, and the strength of binding increases with the length of the CAG tract [91]. In postautopsy human brains and mouse tissues, the activated form of PKR (phospho-PKR) was detected, and the increased immunoreactivity was more pronounced in areas affected by the disease. Strong induction of phospho-PKR in hippocampal neurons was also observed in another study of brain tissues from HD patients [92]. Interestingly, in the FXTAS caused by the expansion of CGG repeats in the 5′ UTR of the* FMR1* gene, RNA hairpins were not shown to activate PKR* in vitro* or* in vivo* [93].

There is no direct evidence that other cellular sensors of dsRNA are activated in cells expressing mutant transcripts; however, Toll-like receptors are identified as potential sensors involved in self-RNA recognition [94, 95].

Indirect evidence of immune system activation by toxic RNA originates mainly from studies of noncoding repeat expansion diseases, such as DM1, DM2, FXS, and FXTAS. Rhodes et al. demonstrated the upregulation of interferon-regulated genes and genes associated with the response to dsRNA as well as the innate immune response in lens epithelium samples obtained from DM1 and DM2 cataracts patients [96]. Many of these genes were dysregulated in both types of DM, suggesting a common causative mechanism. The authors hypothesized that toxic dsRNAs containing expanded CUG and CCUG repeats in DM1 and DM2, respectively, serve as triggers of the interferon response. However, the direct activation of cellular RNA sensors as well as the main elements of the related signaling pathways has not yet been shown. Transcriptional dysregulation of genes involved in the immune response was also detected in the blood of FXTAS carriers [97].

In addition to transcriptional dysregulation, elevated plasma levels of TNF-*α*, IL-6 [98, 99], and IL-1*β* cytokines [99] were demonstrated in DM1 patients. The TNF-*α* levels directly correlated with the length of the CTG expansion and were significantly associated with the disease stage [99]. Interestingly, a later study suggested that elevated levels of TNF-*α* might result from CUGBP1-mediated increased stability of the TNF-*α* mRNA in skeletal muscles and not from a response of immune cells to the disease [100]. CUGBP1 function is affected in DM1, and depletion of CUGBP1, which regulates the stability of the TNF-*α* mRNA, may explain the elevated levels of serum TNF-*α* that is observed in DM1 patients.

Plasma cytokine and chemokine profiles were also studied in FXS patients [101] and* FMR1* knockout mice [102]. The IL-1*α* cytokine level was elevated, the IL-6 level was unchanged, and the RANTES and IP-10 levels were decreased [101]. Different results were obtained when premutation carriers (FXTAS) were studied [103, 104]. The level of the anti-inflammatory cytokine IL-10 in the supernatant of PBMCs derived from premutation carriers was elevated, was correlated with the number of CGG repeats, and was observed before the appearance of the classical neurological symptoms. In another study, decreased cytokine production was observed in the blood cells of FXTAS female carriers and in the splenocytes of* FMR1* knockin mice, and this effect was associated with increased CGG repeat length [104]. These findings suggest that the role of toxic RNA and the immune system in the pathogenesis of FXS and FXTAS is more complex and requires further studies.

## 4. Therapeutic Strategies

Although no causal therapy is currently available and only symptomatic treatment is offered to patients, many therapeutic approaches are being tested to reverse or slow down the progression of the disease. These approaches vary depending on the target, strategy used, delivery method, and experimental model. Taking advantage of the knowledge concerning the pathogenic mechanisms of triplet repeat expansion diseases, the most direct therapeutic strategies target toxic transcript and protein. Antisense oligonucleotides and RNA interference triggers, such as short interfering RNA (siRNA), vector-based short hairpin RNA (shRNA), and artificial miRNA (shmiR), are widely used to silence the expression of mutant and normal/mutant genes in an allele-selective or non-allele-selective strategy, respectively (described in [105–107]). The main issues being explored concern the dose, delivery, and distribution of the therapeutic molecules in the brain, duration of silencing effect, and safety issues [108–110]. In a preclinical study, McBride et al. demonstrated that artificial miRNA molecules delivered into the brain of a human nonprimate model with the use of a viral vector (AAV2/1) were safe and effective [111]. Partial non-allele-specific reduction of* HTT* expression (45%) in the putamen of rhesus macaque is well tolerated until 6 weeks after injection. No signs of local or peripheral inflammation were observed by analysis of reactive microglia or proinflammatory cytokine expression (IL-1*β*, TNF-*α*), which would be predicted to increase if HTT reduction induced neural toxicity. As peripheral tissues are also affected in HD, systemic delivery of artificial miRNAs (AAV serotype 9) targeting mutant huntingtin was recently proposed [112]. RNA interference triggers significantly reduced mutant HTT expression in multiple brain regions and peripheral tissues, thus preventing atrophy and inclusion formation in key brain regions, as well as weight loss in transgenic mice with HD.

Targeting inflammatory pathways and modulating their sensor-transducer-effector functions might be effective in preventing disease progression rather than in reversing the existing pathology. As immune effects manifest both in the CNS and periphery and because there is currently a lack of clarity as to the starting point of the immune cascade, anti-inflammatory therapies will have to target both destinations. On the other hand, suppressing innate immune function may act as a double-edged sword by creating a risk of infections. Thus, selective and fine-tuned therapeutic approaches are required.

Various drugs have been tested to inhibit inflammation pathways in neurodegenerative diseases. Antiapoptotic therapeutic strategies tested in mouse models of polyQ diseases include the inhibition of caspase function [113], inhibition of mitochondrial release of the cytochrome complex, which acts as an activator of the apoptotic pathway [114], and modulation of the initiation of the apoptotic signal [115]. Promising results were obtained with the use of minocycline, which is a second-generation tetracycline that inhibits microglia activation and acts as a caspase inhibitor, thus modulating apoptosis. Therapeutic approaches using minocycline in mouse models of polyQ diseases are described in detail in a review by Switonski et al. [116]. Caspase 1 inhibition by minocycline slows degeneration and disease progression in mouse models of HD [40]. In another study, minocycline delayed disease progression in a R6/2 mouse model of HD by inhibiting caspase-1 and caspase-3 mRNA upregulation and decreasing inducible nitric oxide synthetase activity [42]. However, subsequent studies did not confirm these results and showed no change in survival or even the toxicity of minocycline at higher doses [117, 118].

There are also reports, mostly for HD, describing promising results with the use of cytokine inhibitors. Activation of cannabinoid receptor 2 (CB_2_) decreases inflammatory responses and the production of proinflammatory cytokines and is protective in mouse models of neurodegenerative diseases, including multiple sclerosis, ALS, and Parkinson's disease. CB_2_ receptor levels are elevated in postmortem HD brains and mice models [119, 120], and treatment of the R6/2 HD mouse model with a CB_2_ receptor agonist suppresses neurodegeneration by regulating the IL-6 level in the blood [120]. This effect was even observed in the late stages of the disease and was further confirmed using IL-6-neutralizing antibodies. These findings support the link between peripheral immune cell signaling and neurodegeneration in HD [120]. Surprisingly, elevated cytokine levels may be normalized by silencing mutant huntingtin using RNAi. The authors demonstrated that mutant huntingtin induces the transcriptional changes and NF*κ*B pathway dysregulation that results in elevated cytokine levels. After treating human primary macrophages (LPS stimulated) with anti-HTT-siRNA the huntingtin level decreased, and the IL-6, IL-8, and TNF-*α* levels were significantly reduced [51].

Another study demonstrated that transplantation of wild-type bone marrow cells into HD mice ubiquitously expressing full-length huntingtin (YAC128 and BACHD mice) normalizes the elevated levels of serum cytokines and chemokines, including IL-6, IL-10, CXCL1, and IFN*γ* [36]. Furthermore, peripheral administration of a kynurenine 3-monooxygenase (KMO) inhibitor decreases microglial activation, extends life span and improves the phenotype of HD mice [121].

Although immune system activation is probably not a main pathogenic factor in TREDs, a number of studies show that it may be a good therapeutic target. The tested drugs improve the phenotypes of treated animals by normalizing cytokine levels, slowing neurodegeneration and disease progression, and extending life span, which bodes well for results in human trials.

## 5. Summary

It seems that the inflammation observed in repeat expansion diseases may actively influence the progression of the disease. However, the normal functions of proteins in polyQ diseases may modulate the immune response because these proteins are involved in various processes, including transcription regulation, ubiquitin-mediated proteolysis, alternative splicing, and chromatin remodeling. These different functions might explain the different results obtained for specific polyQ diseases. The results presented in this review indicate that both toxic factors, that is, protein and RNA, may act to trigger inflammatory pathways and neurodegeneration. Further understanding of the role of inflammation in the pathogenesis of TREDs may allow for the design of better therapeutic approaches, slowing disease progression and improving the life of patients.

## Supplementary Material

Supplementary Table 1: presents the function of the proteins, the main sites of pathogenesis, and the characteristics of the clinical features of TREDs.
Supplementary Table 2: presents published data concerning immunological effects observed in different cell/animal models as well as in TREDs patients.
