# Supplementary material for: The Role of the Immune System in Triplet Repeat Expansion Diseases
Source: Mediators Inflamm. 2015 Mar 22;2015:873860. doi: 10.1155/2015/873860 (PMC4385693; doi:10.1155/2015/873860)
Supplement: Supplementary file 1 — Supplementary Table 1: presents the function of the proteins, the main sites of pathogenesis, and the characteristics of the clinical features of TREDs. Supplementary Table 2: presents published data concerning immunological effects observed in different cell/animal models as well as in TREDs patients. [file 873860.f1.pdf]

DISEASE	GENE	TYPE OF REPEATS	REPEATS LOCALIZATION	NUMBER OF REPEATS (NORMAL/ *MUTANT*)	PROTEIN PRODUCT OF THE GENE /FUNCTION	MAIN SITE OF PATHOGENESIS	CLINICAL FEATURES
HD<br>*Huntington's disease*	*HTT*	CAG	ORF	9-36/*37-160*	huntingtin/ embryonic development, neurogenesis	striatum, globus pallidus, substantia nigra	chorea, dystonia, incoordination, cognitive decline, and behavioral difficulties
DRPLA<br>*dentatorubral-pallidoluysian atrophy*	*ATN1*	CAG	ORF	9-36/*38-62*	atrophin-1 / transcription regulation	globus pallidus, subthalamic nucleus, dentate nucleus, white matter	myoclonic epilepsy, dementia, ataxia and choreoathetosis
SBMA<br>*X-linked spinal and bulbar muscular atrophy*	*AR*	CAG	ORF	9-36/*38-62*	androgen receptor / steroid-hormone activated transcription	spinal anterior horn, facial nucleus, skeletal muscle	male pseudohermaphroditism, hypospadias and cryptorchidism, spinal and bulbar muscular atrophy
SCA1<br>*spinocerebellar ataxia type 1*	*ATXN1*	CAG	ORF	6-44/*39-82*	ataxin-1/transcription regulation, alternative splicing	Purkinje cells, dentate nucleus, brainstem, spinal cord	cerebellar ataxia, ophthalmoplegia, pyramidal and extrapyramidal signs, peripheral neuropathy, and dementia
SCA2<br>*spinocerebellar ataxia type 2*	*ATXN2*	CAG	ORF	13-31/*32-500*	ataxin-2 / mRNA maturation and translation, stress-granule formation, endocytosis, Ca-mediated signaling	cerebellum (Purkinje cells), pons, inferior olives, thalamus, substantia nigra	cerebellar ataxia, ophthalmoplegia, pyramidal or extrapyramidal signs, mild dementia, and peripheral neuropathy
SCA3/MJD<br>*spinocerebellar ataxia type 3/ Machado-Joseph disease*	*ATXN3/ MJD*	CAG	ORF	up to 44/ *52-86*	ataxin-3 / ubiquitin-mediated proteolysis, chromatin remodeling	substantia nigra, cranial nerve motor nuclei, striatum	ataxia, spasticity and ocular movement abnormalities
SCA6<br>*spinocerebellar ataxia type 6*	*CACNA1A*	CAG	ORF	4-18/*19-33*	alpha-1A calcium channel protein/ calcium-dependent processes	Purkinje cells, dentate nucleus, inferior olive	progressive cerebellar ataxia of the limbs and gait, dysarthria, nystagmus and mild vibratory and proprioceptive sensory loss
SCA7<br>*spinocerebellar ataxia type 7*	*ATXN7*	CAG	ORF	4-35/*37-306*	ataxin-7 / transcription regulation	Purkinje cells, pons, dentate nucleus, inferior olive, retina	progressive cerebellar ataxia with pigmentary macular degeneration, ophthalmoplegia, pyramidal or extrapyramidal signs, deep sensory loss or dementia

SCA17/ HDL4 *spinocerebellar ataxia type 17/ Huntington's disease-like 4*	*TBP gene*	CAG	ORF	25-44/*47-63*	TATA box-binding protein / transcription regulation	Purkinje cells, striatum, cerebral cortex	ataxia, pyramidal and extrapyramidal signs, cognitive impairments, psychosis, and seizures
SCA12 *spinocerebellar ataxia type 12*	*PPP2R2B*	CAG	5'UTR	7-32/51-78	brain-specific regulatory subunit of the protein phosphatase PP2A/cell growth and division, muscle contraction, and gene transcription	cerebellum and cerebrum	upper extremity tremor, head tremor, gait ataxia, dysmetria, dysdiadokinesis, hyperreflexia, paucity of movement, abnormal eye movements
SCA8 *spinocerebellar ataxia type 8*	*ATXN8*	CAG/ CTG	3'UTR	15-50/ *71-1300*	ataxin-8/unknown	cerebellum (Purkinje cells), pons	spastic and ataxic dysarthria, nystagmus, limb and gait ataxia, limb spasticity, and diminished vibration perception
HDL2 *Huntington's disease-like 2*	*JPH3*	CTG	ORF/ 3'UTR	6-28/*over 41*	junctophilin-3/mediate cross talk between cell surface and intracellular ion channels	cerebrum and caudate	clinically similar to Huntington disease
DM1 *myotonic dystrophy type 1*	*DMPK*	CTG	3'UTR	5-37/ *50-10000*	dystrophia myotonica protein kinase/unknown	distal muscles of the extremities	myotonia, muscular dystrophy, cataracts, hypogonadism, frontal balding, and ECG changes
DM2 *myotonic dystrophy type 1*	*ZNF9*	CCTG	intron	up to 30/ 75-11000	ZNF9 protein/ RNA-binding protein	skeletal muscle, lens, and heart	muscle pain and stiffness, progressive muscle weakness, myotonia, male hypogonadism, cardiac arrhythmias, diabetes, and early cataracts
FXTAS *fragile X-associated tremor ataxia syndrome*	*FMR1*	CGG	5'UTR	6-40 / *55-200*	Fragile X mental retardation protein/germ cell proliferation	cerebrum, cerebellum, and cerebral cortex	progressive intention tremor, parkinsonism, cognitive decline, generalized atrophy on MRI, and impotence
FXS *fragile X syndrome*	*FMR1*	CGG	5'UTR	5-44/*over 200*	Fragile X mental retardation protein/germ cell proliferation	posterior cerebellar vermis, amygdala, and superior temporal gyrus	severe mental retardation, macroorchidism, and distinct facial features, including long face, large ears, and prominent jaw
FRAXE *fragile XE syndrome*	*FMR2*	CCG	5'UTR	4-39/*200-900*	AF4/FMR2 family member 2 /unknown	hippocampus and the amygdala	mental retardation and behavioral abnormalities
FRDA1 *Friedreich's ataxia*	*FXN*	GAA	intron	5-30/ *70-over 1000*	frataxin/iron-sulfur biogenesis and heme biosynthesis	spinocerebellar tracts, dorsal columns, pyramidal tracts, cerebellum and medulla	progressive gait and limb ataxia with associated limb muscle weakness, absent lower limb reflexes, extensor plantar responses, dysarthria, and decreased vibratory sense and proprioception

Supplementary Table 1. Characteristics of diseases caused by simple repeat expansion.

DISEASE	EXPERIMENTAL MODEL	OBSERVED EFFECTS	REFERENCE
HD	serum from HD patients	Elevated levels of IgA, sTNF-R, sIL-R, neopterin, complement component C3, decreased level of tryptophan	Leblhuber et al. 1998
	Brain samples of HD patients	Complement activation (Complement component C3 and C9 expression in microglia	Singhrao et al. 1999
	lymphoblasts from HD patients	lymphoblasts exhibit more apoptotic vulnerability through caspase-3 and caspase-9 pathway activation by staurosporine; not observed in SCA1 patients	Sawa et al. 1999
	mouse model of HD (R6/2)	Caspase-1 and caspase-3 activation, increased iNOS expression	Chen et al. 2000
	Caudate of HD patients	Increased iNOS expression	Chen et al. 2000
	Brain samples of HD patients	Increased phospho-PKR level in areas of brain	Peel et al. 2001
	Brain samples of mouse model of HD (YAC)	Increased phospho-PKR level in areas of brain	Peel et al. 2001
	Brain samples of HD patients	Increased level of phospho-PKR in neurons	Bando et al. 2005
	Blood cells from HD patients	Differences in expression of many genes	Borovecki et al. 2005
	Blood cells from HD patients	Not significant changes in gene expression	Runne et al. 2007
	Plasma from HD patients	Increase of IL-6 level	Dalrymple et al. 2007
	Plasma from mouse model of HD (R6/2)	Increase of IL-6 level	Dalrymple et al. 2007
	In vivo brain imaging of HD patients	Microglial activation	Tai et al. 2007
	Brain samples of mouse model of HD (R6/2)	Increased levels of neurotoxic kynurenine metabolites	Giorgini et al. 2008
	plasma from HD patients (premanifest to moderate)	Increased levels of cytokines IL-6, IL-8, IL-4, IL-10, TNF-α, no difference in immunoglobulin levels	Bjorkqvist et al. 2008
	monocytes from HD patients	excess IL-6 production after stimulation with IFN-γ and LPS	Bjorkqvist et al. 2008
	macrophages from mouse model of HD (YAC128, R6/2)	excess IL-6 production after stimulation with IFN-γ and LPS	Bjorkqvist et al. 2008
	striatal tissue from HD patients	Increased levels of IL-6, IL-8, TNF-α	Bjorkqvist et al. 2008
	Serum from mouse model of HD (R6/2, *Hdh*$^{150Q/150Q}$, YAC128)	Increased levels of IL-6, IL-10, IL-8 homologue, IL-12p70, IL-1β (pattern dependent on mice model)	Bjorkqvist et al. 2008
	Stable and inducible neuro 2a cel line expressing tNhtt containing polyQ tract	Increase level of inflammatory genes (KC, MCP-1, LIX, IL-6, TIMP), c-jun, p-c-jun, p38 and ERK1/2, decreased levels of TNF-α and IL-10, downregulation of NF-κB	Godavarthi et al. 2009
	Brain samples of mouse model of HD (R6/2)	No differences in expression of various inflammatory molecules, no apparent inflammation	Godavarthi et al. 2009
	plasma from HD patients	altered chemokine profile (eotaxin-3, MIP-1ß, eotaxin, MCP-1, MCP-4)	Wild et al. 2011
	blood from HD patients	increasing with disease progression level of mutant HTT in leukocytes	Weiss et al. 2012
	cell culture from mouse model of HD (YAC128 and BACHD)	decrease in microglia and macrophages migration to chemotactic stimuli	Kwan et al. 2012
	microglial cell lines (BV2 cells)	decrease in microglia and macrophages migration to chemotactic stimuli	Kwan et al. 2012
	Mouse model of HD (BACHD)	functional impairment of microglia	Kwan et al. 2012
	blood from HD patients	decrease in monocytes and macrophages migration	Kwan et al. 2012
	Mouse model of HD (BACHD and R6/2)	peritoneal macrophage infiltration in response to an inflammatory stimulus is strongly impaired, also found in other cells of the myeloid lineage (BACHD only)	Kwan et al. 2012a
	Mouse model of HD (YAC128)	elevated levels of IL-6, CXCL1 (mouse homolog of IL-8), IFN-γ and IL-10; not observed in BACHD mice	Kwan et al. 2012b
	Mouse model of HD (*Hdh*^{150Q}, R6/2, N171-82Q)	LPS stimulation induced higher levels of TNF-α, IL-1β in *Hdh*^{150Q} *and* R6/2 mouse models in brain, serum and liver; in R6/2 mice there was increase of NF-κB levels in actrocytes but not in microglia; in N171-82Q model no chronic inflammation was observed	Hsiao et al. 2013
	Astrocytes from mouse model of HD (R6/2)	Cells were more susceptible to LPS stimulation, higher phosporylation level of NF-κB inhibitor kinase (IKK), co-cultured activated astrocytes from R6/2 produced more damaging effect on neurons	Hsiao et al. 2013
	Astrocytes from HD patients	Activation of NF-κB (nuclear localization)	Hsiao et al. 2013
	monocytes and macrophages from HD patients	Elevated production of IL-6, TNF-α after stimulation; IL-8, IL-10, IL-12 levels were not changed in monocytes; elevated levels of IL-8 and TNF-α in macrophages; interaction between IKK complex and HTT; significantly upregulated genes (e.g. TLR2, IL10)	Trager et al. 2014

DISEASE	EXPERIMENTAL MODEL	RESULT	REF.
SCA1	Mouse model of SCA1 (DTg)	Strong astrocytosis (analyzed by GFAP level), $EAAT_1$ rearrangement	Giovannoni et al. 2007
SCA3	Rat cell model of SCA3 (CSM14.1 expressing human ataxin-3)	Increase of MMP-2, APP, Fit-1S, SDF1α levels	Evert et al. 2001
	Brain samples of HD patients	Increase of MMP-2, Aβ, IL-1ra, IL-1β, SDF1, CD68 levels in pons	Evert et al. 2001
	Rat cell model of SCA3 (CSM14.1 expressing human ataxin-3)	Increase of expression of many genes, among them inflammatory cytokines (Fit-1S, IL-1ra, IL-6, C/EBPβ and C/EBPδ).	Evert et al. 2003
	Brain samples material from SCA3 patients	Increase of IL-6, C/EBPδ levels in pons, same level of C/EBPβ	Evert et al. 2003
	Brain samples from SCA3 patients	Increase of IL-1β, IL1-ra, IL-6 and C/EBPβ (mainly or only in pontine); increase of CD68+ microglia in pons and dentate nucleus, pallidum, oculomotor nucleus and the substantia nigra	Evert et al. 2006
	Mouse model of SCA3	Reduced immune defence (bacterial infections), increased unspecific immune response (granulocytes), decreased specific immune response (B cells), significant differences in levels of inflammatory markers were not observed; age-dependent increased number of activated microglia in pontine (Iba1 staining), no significant increase of reactive astrocytes were observed	Hubener et al. 2012
DM1	Serum from DM1 patients	Elevated levels of IL-6 and TNF-α	Johansson et al. 2000
	in vitro experiment	PKR binds to CUG repeats and undergo autophosphorylation	Tian et al. 2000
	Plasma from DM1 patients	Elevated levels of IL-6, IL-1β and TNF-α	Mammarella et al. 2002
	C2C12 cells with DMPK(960 CUG repeats) expression	TNF mRNA stabilization	Zhang et al. 2008
	DM1 myoblasts	Elevation of PKR level	Huichalaf et al. 2010
	Lens epithelial cells from DM1 patients	Upregulation of interferon-regulated genes and immune response genes	Rhodes et al. 2012
DM2	Lens epithelial cells from DM2 patients	Upregulation of interferon-regulated genes and immune response genes	Rhodes et al. 2012
FXS/FXTAS	HeLa cells transfected with in vitro sythesized CGG RNA or isogenic line expressing CGG RNA	No upregulation of PKR (no phosphorylation of PKR substrate eIF2α)	Handa et al. 2003
	Fmr1 Knock-out mouse	Reactive astrocytes in brains (elevated GFAP expression); no differences in serum levels of cytokines; no differences in IL-6 and TNF-α levels in microglia after LPS induction	Yuskaitis et al. 2010
	Plasma from FXS patients	Elevated level of IL-1α, decreased levels of RANTES and IP-10, no difference in IL-6 level	Ashwood et al. 2010
	PBMC from pre-mutation patients (FXTAS)	Elevated IL-10 level (correlated with CGG repeats length)	Marek et al. 2012
	Blood from premutation carriers (PBLs, monocytes)	Decrease in cytokine levels (associated with CGG length), T cells showed decrease in cell surface markers	Careaga et al. 2014
	FMR1 Knock-in mouse (splenocytes)	Decrease in cytokine levels (e.g. IL-6, IL-13, IL-17)	Careaga et al. 2014
	Blood from premutation carriers	Gene deregulation, including genes involved in inflammatory response and immune response	Mateu-Huertas et al. 2014

Supplementary Table 2 Experimental verification of inflammation in simple repeats expansion diseases.
